# The Aged Retinal Pigment Epithelium/Choroid: A Potential Substratum for the Pathogenesis of Age-Related Macular Degeneration

**DOI:** 10.1371/journal.pone.0002339

**Published:** 2008-06-04

**Authors:** Huiyi Chen, Bin Liu, Thomas J. Lukas, Arthur H. Neufeld

**Affiliations:** Forsythe Laboratory for the Investigation of the Aging Retina, Department of Ophthalmology, Northwestern University School of Medicine, Chicago, Illinois, United States of America; University of Oldenburg, Germany

## Abstract

**Background:**

Although the statement that age is the greatest risk factor for Age-related macular degeneration (AMD) is widely accepted, the cellular and molecular explanations for that clinical statement are not generally known. A major focus of AMD research is the retinal pigment epithelium (RPE)/choroid. The purpose of this study was to characterize the changes in the RPE/choroid with age that may provide a background for the development of AMD.

**Methodology/Principal Findings:**

We compared the transcriptional profiles, key protein levels and histology of the RPE/choroid from young and old mice. Using three statistical methods, microarray data demonstrated marked changes in the old mouse. There were 315 genes differentially expressed with age; most of these genes were related to immune responses and inflammatory activity. Canonical pathways having significant numbers of upregulated genes in aged RPE/choroid included leukocyte extravasation, complement cascades, natural killer cell signaling and IL-10 signaling. By contrast, the adjacent neural retina showed completely different age-related changes. The levels of proteins that participate in leukocyte extravasation and complement pathways were consistently increased in the normal, aged RPE/choroid. Furthermore, there was increased gene expression and protein levels of leukocyte attracting signal, chemokine ligand 2 (Ccl2) in aged RPE/choroid. In old animals, there was marked extravasation and accumulation of leukocytes from the choroidal circulation onto Bruch's membrane and into the RPE.

**Conclusions/Significance:**

These phenotypic changes indicate that the RPE/choroid in the normal, old mouse has become an immunologically active tissue. There are signals from the normal, aged RPE/choroid which recruit leukocytes from the circulation and activate the complement cascade. These age-related changes that occur in the RPE/choroid with age, to the extent that they occur in the human retina, may provide the background for an error in regulation of immunological activity to cause AMD to appear in an elderly individual.

## Introduction

Although the statement that age is a risk factor for many adult human diseases is widely accepted, the cellular and molecular explanations for that clinical statement are not generally known [1], [2]. We believe that older individuals have underlying changes in specific tissues that increase the susceptibility of the tissues to causative disease processes and/or contribute to progression of the age-related disease. Thus, certain age-related changes can be identified as susceptibility factors and may occasionally be manifested clinically as risk factors. The key to identifying these underlying factors is to characterize the phenotype of the normal, aged tissue.

We hypothesize that normal aging of the retinal pigment epithelium (RPE)/choroid provides a background for the development of age-related macular degeneration (AMD). The RPE, which is adjacent to the photoreceptors and rests on Bruch's membrane, phagocytoses and digests the distal parts of the outer segments of the photoreceptors. The choroid, on the other side of Bruch's membrane, supplies oxygen and nutrients to the RPE and photoreceptors. The early stage of dry AMD is characterized by the presence of drusen in the macula between the RPE and Bruch's membrane. Drusen diminishes diffusion from the choroidal circulation to the retina, causing adverse effects on both the RPE and photoreceptors [3], [4]. Many laboratories studying the components of drusen have shown that drusen has a complex protein composition that includes immunoglobulins, activated complement components and complement regulators from the choroidal circulation [4]–[6], and lipids, intracellular proteins and cytosolic stress response proteins from RPE cells [6]. However, the etiology of drusen and why the presence of drusen increases with age are not completely understood.

AMD is likely to be a multi-factorial disease. Human genetic studies have identified genes, e.g. ABCA4 [7], CX3CR1 [8], ELOVL4 [9], APOE [10], [11] and MMP9 [12], the mutations of which are associated with AMD. Recently, variants of CFH (complement factor H) [9], [13], LOC387715/HTRA1 [14]–[16] and BF/C2 (complement factor B/ complement component 2) [17], [18] have been highlighted as major loci contributing to AMD [19], [20]. In human RPE/choroid, there are differentially expressed genes related to inflammation in the macular region compared to extramacular regions [21]. Whereas several genes are associated with AMD and more are likely to be identified, the genetic findings do not provide an explanation for the onset of AMD late in life.

To test our hypothesis that there are underlying changes in the RPE/choroid with age that provide a background for the development of AMD, we compared gene expression profiles, key protein levels and cell markers in the RPE/choroid of young and old mice. Our results indicate that in the old mouse, the RPE/choroid has become an immunologically active tissue. We believe that these marked differences with age in the RPE/choroid, if similar changes occur in the human retina, contain the underlying cellular and molecular activities for the development of AMD.

## Results

### Comparison of functional categories of altered gene expression with age

**Figure 1:**
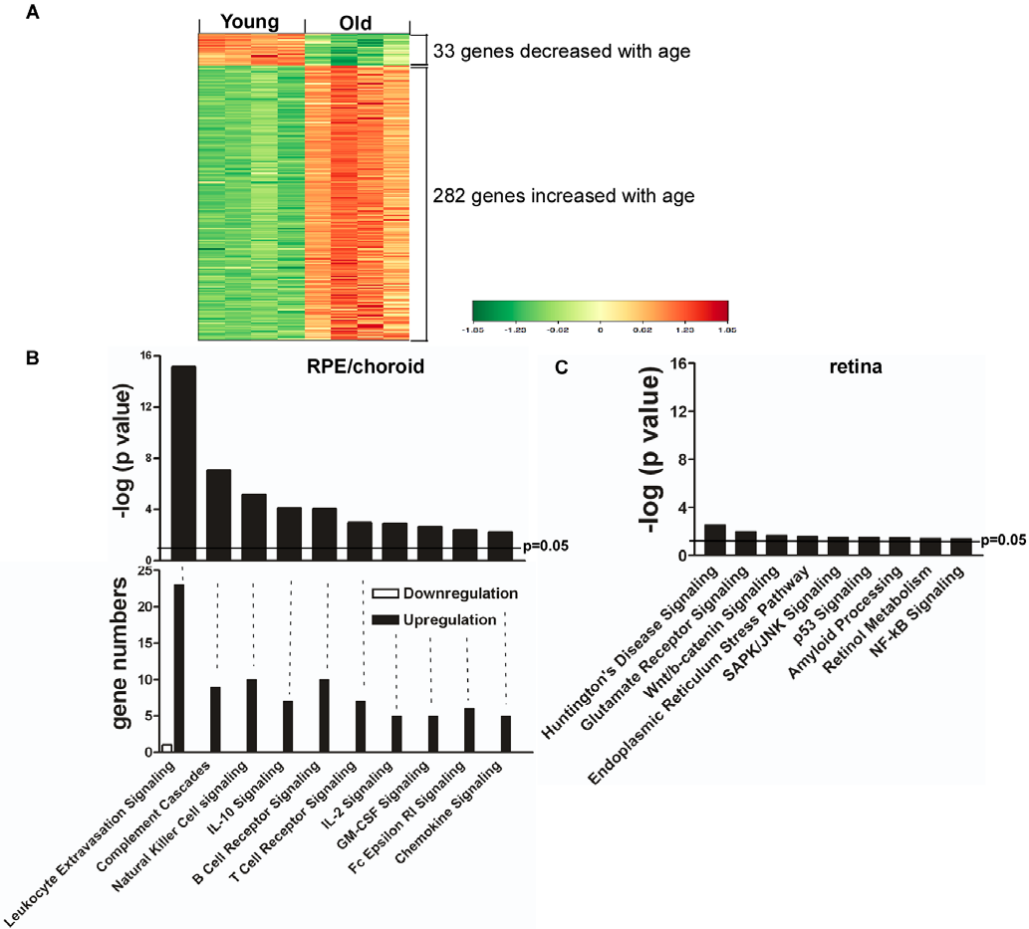
Changes in gene expression in RPE/choroid from old animals. (A) The transcriptional profiles of the normal RPE/choroid from young and old mice were analyzed by hierarchical clustering of 315 differentially expressed, age-regulated genes by Limma analysis. Standardized expression values of genes are displayed according to the color scale, in which red represents above average expression and green represents below average expression. Absolute fold changes of individual genes are shown in Table S1 online. (B) Relative changes in canonical pathways in RPE/choroid from old animals. The upper figure shows the –log (p value) of the first 10 canonical pathways which changed significantly in RPE/choroid from old animals. The horizontal line represents the threshold of p which is equivalent to p = 0.05. Bars above the line indicate p<0.05. The lower part of the figure shows the number of differentially expressed genes in each pathway. (C) The –log (p value) of the 9 canonical pathways which changed significantly in neural retina from old animals.

The microarray data from RPE/choroid have been deposited in NCBIs Gene Expression Omnibus (GEO, http://www.ncbi.nlm.nih.gov/geo/) and are accessible through GEO Series accession number GSE10965. By comparing RPE/choroid tissues from young and old animals, the analyses of our microarray data showed consistent results and distinctive transcriptional profiles. There were 315 differentially expressed genes from the analysis using Limma (Table S1), 300 from DNA-Chip Analyzer (Dchip) and 309 from Significance Analysis of Microarrays (SAM). 227 genes were in common for the three analytical methods. To identify the distinctive transcriptional profile, we performed hierarchical clustering to analyze the 315 differentially expressed genes from Limma analysis. A clustering map is shown in Figure 1 A, comparing the expression of 315 genes in the RPE/choroid from young and old animals and clearly demonstrating marked differences. Among the 315 genes, 33 genes were decreased and 282 genes were increased with age.

Global functional analyses and canonical pathway analyses using Ingenuity Pathway Analysis of the 315 differentially expressed genes (Limma) yielded distinct categories and pathways related to immune responses and inflammatory activities. There were 16 canonical pathways that were significantly upregulated in aged RPE/choroid. The first 10 canonical pathways, ordered by significance (Fig. 1 B) are: (1) leukocyte extravasation signaling; (2) complement cascades; (3) natural killer (NK) cell signaling; (4) IL-10 signaling; (5) B cell signaling; (6) T cell signaling; (7) IL-2 signaling; (8) GM-CSF (granulocyte monocyte colony stimulating factor) signaling; (9) Fc Epsilon RI signaling; and (10) chemokine signaling. Nearly all the differentially expressed genes in these pathways were upregulated (Fig. 1 B). Using the 300 genes from Dchip and the 309 genes from SAM showed the same canonical pathways when analyzed using Ingenuity Pathway Analysis. We interpreted these results to indicate that there were increased immunological and inflammatory activities in the RPE/choroid of old animals. By contrast, the adjacent neural retina showed completely different age-related changes in its transcriptional profile. Of the 9 canonical pathways that showed significant changes in neural retina with age, most were related to stress responses and apoptosis (Fig. 1 C), but no immunological or inflammatory pathways were upregulated.

### Confirmation of altered gene expression by PCR

**Figure 2:**
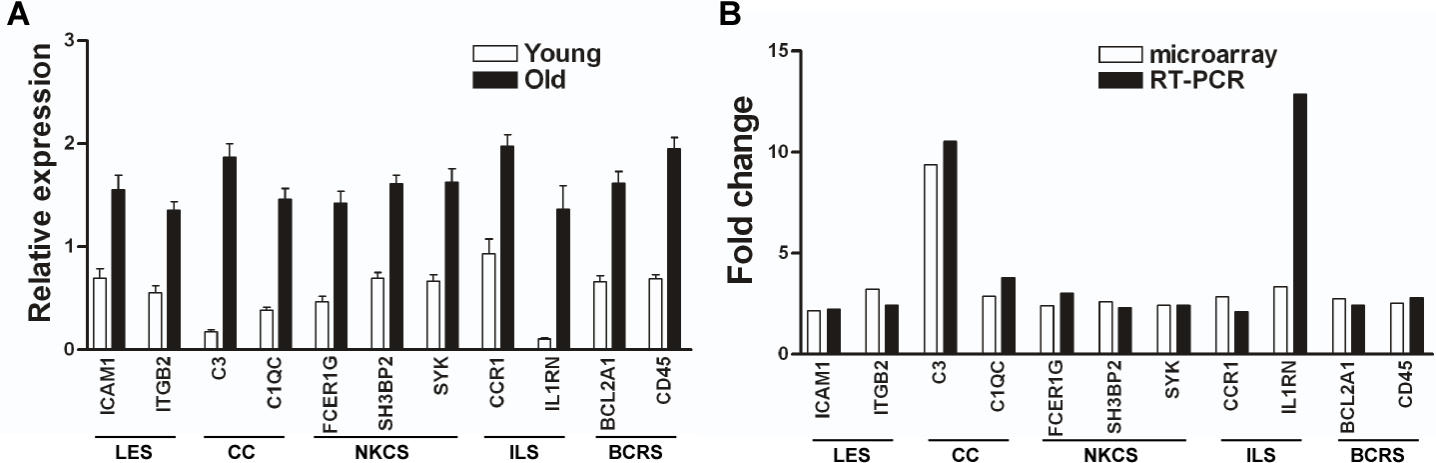
Real time RT-PCR confirmation of microarray results. (A) Relative mRNA expression levels of selected genes from the first 5 canonical pathways (see Fig. 1B) in RPE/choroid from young and old animals. (B) Comparison of the fold changes of the same selected genes determined by microarray analysis and real-time RT-PCR. LES, leukocyte extravasation signaling; CC, complement cascade; NKCS, natural killer cell signaling; ILS, IL-10 signaling; BCRS, B cell receptor signaling.

To validate the microarray data, we performed quantitative RT-PCR on 2–3 functionally important genes from each of the first 5 significantly changed canonical pathways. RT-PCR confirmed that all of the increased genes chosen from the microarray results were significantly increased (Fig. 2 A). Moreover, the fold changes of old vs. young gene expression levels were consistent, comparing the microarray and RT-PCR data (Fig. 2 B). Only one gene, IL1RN, showed a higher fold increase using RT-PCR than predicted from microarray data (12.9 fold vs. 3.2 fold).

### Putative altered functional pathways with age

**Figure 3:**
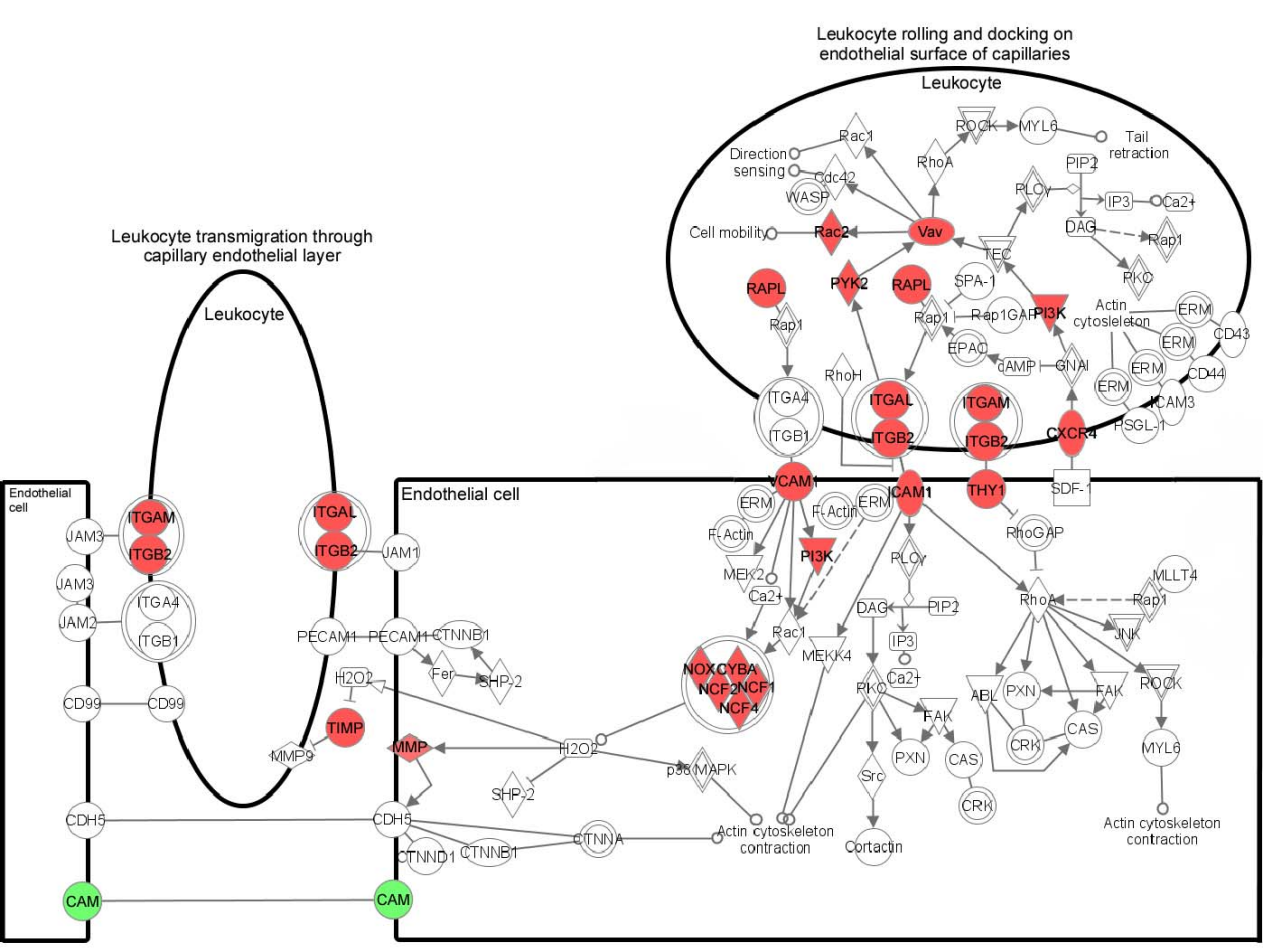
Pathway diagram showing the molecules involved in leukocyte extravasation signaling and their interaction. Color nodes: genes that changed significantly in RPE/choroid from old animals with a fold change >2. Red: upregulation; green: downregulation. The diagram was modified from Ingenuity Pathway Analysis (Ingenuity® Systems).

The pathway with changes of the highest significance was leukocyte extravasation signaling (p<10^−16^). There were 24 genes in this pathway that significantly changed in the aged RPE/choroid by Limma analysis and almost all showed significant changes by Dchip and SAM analyses (Table 1; Fig. 1 B and 2 A). Expression of 23 genes was increased, suggesting high leukocyte extravasation activity in the RPE/choroid of old animals. The upregulated genes are involved in leukocyte rolling and docking on endothelial surfaces of capillaries and leukocyte transmigration through capillary endothelial layers (Fig. 3). Several membrane receptors that are crucial for interaction between leukocytes and endothelial cells and for enhancing leukocytes recruitment [22]–[25] showed significantly increased expression in the RPE/choroid of old animals, including: ITGAM (αM-integrin), ITGB2 (β2-integrin), ITGAL (αL-integrin) and ICAM1 (intercellular adhesion molecule 1). In addition, expression of matrix metallproteases, that are important effectors of inflammatory processes and also essential for leukocyte extravasation and migration [26], [27], was also significantly increased, notably: MMP3 (matrix metalloprotease 3) and MMP13 (matrix metalloprotease 13).

**Table 1:** Age-related changes in gene expression in RPE/choroid.

Name	Description	Gene Bank	Fold Change	p value	No.
**Leukocyte Extravasation Signaling**					
CXCR4	chemokine (C-X-C motif) receptor 4	D87747	2.9	0.000242	3
CYBA	cytochrome b-245, alpha polypeptide	AK018713	2.3	5.79E-05	3
CYBB	cytochrome b-245, beta polypeptide	AV373944	2.9	3.30E-05	3
ICAM1	intercellular adhesion molecule 1 (CD54)	BC008626	2.1	6.26E-05	3
ITGAL	integrin, alpha L (antigen CD11A (p180)	BI554446	2.6	0.00041451	3
ITGAM	integrin, alpha M (complement component 3 receptort)	NM_008401	3.4	0.00017275	3
ITGB2	integrin, beta 2 (complement component 3 receptor)	NM_008404	3.2	8.51E-05	3
MMP3	matrix metallopeptidase 3 (stromelysin 1, progelatinase)	NM_010809	12.2	1.85E-05	3
MMP12	matrix metallopeptidase 12 (macrophage elastase)	BC019135	2.1	0.001043	3
MMP13	matrix metallopeptidase 13 (collagenase 3)	NM_008607	9.1	0.00031966	3
MMP19	matrix metallopeptidase 19	NM_021412	2.4	0.0034653	3
NCF1	neutrophil cytosolic factor 1	AI844633	2.5	6.61E-05	3
NCF2	neutrophil cytosolic factor 2	NM_010877	2.1	0.00073191	2
NCF4	neutrophil cytosolic factor 4	NM_008677	2.6	0.00037003	3
PIK3R5	phosphoinositide-3-kinase, regulatory subunit 5	AV230647	3.0	0.00031618	3
PTK2B	PTK2B protein tyrosine kinase 2 beta	NM_172498	2.1	0.00013604	3
RAC2	rho family, small GTP binding protein Rac2	NM_009008	2.4	0.00020253	3
RASSF5	Ras association (RalGDS/AF-6) domain family 5	NM_018750	2.1	0.00058134	3
THY1	Thy-1 cell surface antigen	NM_009382	2.1	0.00023721	2
TIMP1	TIMP metallopeptidase inhibitor 1	BC008107	2.3	0.00039729	3
TIMP4	TIMP metallopeptidase inhibitor 4	BI788452	3.8	3.32E-05	3
VAV1	vav 1 oncogene	NM_011691	2.6	5.35E-05	3
VCAM1	vascular cell adhesion molecule 1	L08431	2.5	5.04E-05	3
CLDN5	claudin 5	NM_013805	−2.1	0.0041074	1
**Complement Cascades**					
C3	complement component 3	K02782	9.4	3.47E-05	3
C1QA	complement component 1, q subcomponent, A chain	NM_007572	2.3	6.61E-05	3
C1QB	complement component 1, q subcomponent, B chain	NM_009777	2.6	5.24E-05	3
C1QC	complement component 1, q subcomponent, C chain	NM_007574	2.9	5.84E-05	3
C1R	complement component 1, r subcomponent	NM_023143	2.5	9.70E-06	3
C1S	complement component 1, s subcomponent	BC022123	2.4	5.24E-05	3
C3AR1	complement component 3a receptor 1	NM_009779	2.1	6.61E-05	3
C4B	complement component 4B	NM_009780	2.3	6.49E-05	3
CFB	complement factor B	NM_008198	2.2	3.73E-05	1
PLAUR	plasminogen activator, urokinase receptor	NM_011113	2.4	0.0055312	2
**NK cell Signaling**					
CER1G	Fc fragment of IgE, high affinity I, receptor for	NM_010185	2.4	3.30E-05	3
FCGR3A	Fc fragment of IgG, low affinity IIIa, receptor (CD16a)	BC027310	2.7	0.0014469	3
LAIR1	leukocyte-associated immunoglobulin-like receptor 1	AV370380?	3.5	0.00030431	3
LCK	lymphocyte-specific protein tyrosine kinase	NM_010693	2.2	0.00045097	2
PIK3R5	phosphoinositide-3-kinase, regulatory subunit 5, p101	AV230647	3.0	0.00031618	3
RAC2	rho family, small GTP binding protein Rac2	NM_009008	2.4	0.00020253	3
H3BP2	SH3-domain binding protein 2	BC010198	2.6	0.00024365	3
SYK	spleen tyrosine kinase	AW907526	2.4	0.00012293	3
YROBP	TYRO protein tyrosine kinase binding protein	NM_011662	2.3	3.30E-05	3
VAV1	vav 1 oncogene	NM_011691	2.6	5.35E-05	3
**IL-10 Signaling**					
ARG2	arginase, type II	NM_009705	2.0	0.0039292	1
CCR1	chemokine (C-C motif) receptor 1	AV231648	2.9	0.00083896	3
CGR2B	Fc fragment of IgG, low affinity IIb, receptor (CD32)	BM224327	3.3	5.79E-05	3
FCGR3	Fc fragment of IgG, low affinity III	NM_010188	2.5	0.00024334	3
HMOX1	heme oxygenase (decycling) 1	NM_010442	2.4	0.00071255	2
IL10RA	interleukin 10 receptor, alpha	NM_008348	2.6	6.15E-05	3
IL1RN	interleukin 1 receptor antagonist	M57525	3.4	6.61E-05	3

Shown are the differentially expressed genes in the 4 most significantly changed pathways in aged RPE/choroid by Limma analysis. Fold changes compare old vs. young. “No.” means the number of statistical methods (Limma, Dchip and SAM) in which each gene appeared changed. See Table S1 online for a complete list of gene expression changes in the RPE/choroid of old animals.

**Figure 4:**
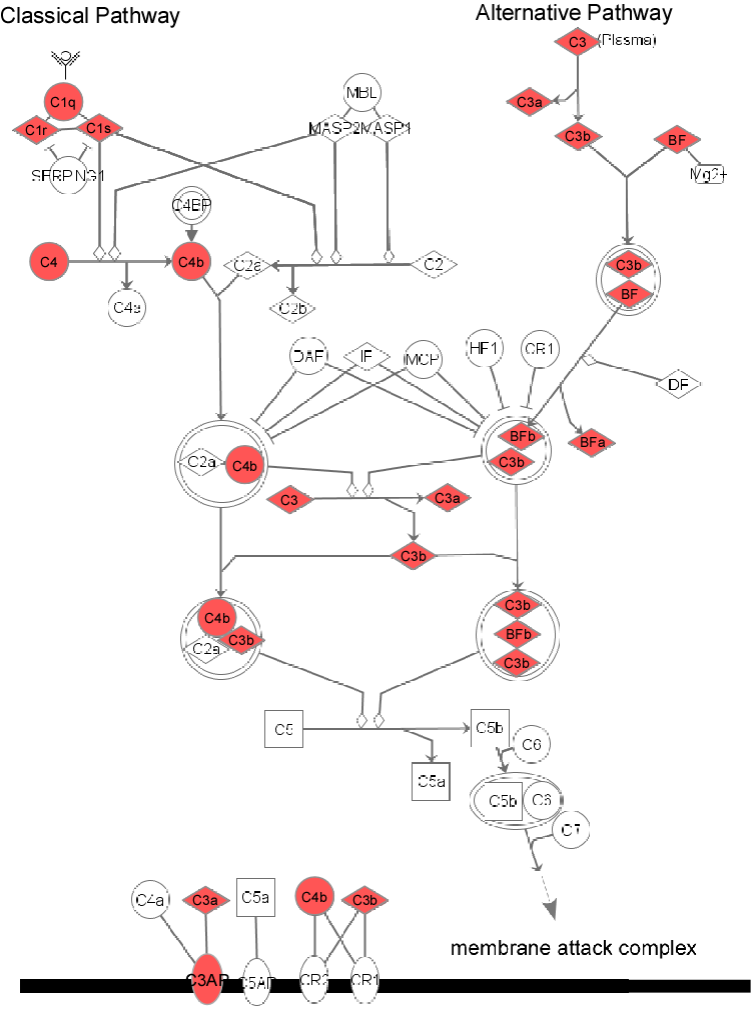
Pathway diagram showing the molecules involved in complement pathway and their interaction. The diagram was modified from Ingenuity Pathway Analysis (Ingenuity® Systems).

Gene expression of members of the complement cascades was upregulated (p<10^−8^) (Table 1; Fig. 1 B and 2 A) in the RPE/choroid of old animals. Nine genes in the complement cascades increased significantly with age (Fig. 4). Most of these genes are involved in activation of either the classical complement pathway or the alternative complement pathway, e.g. C1q (complement component 1, q subcomponent, A chain, B chain and C chain), C1r (complement component 1, r subcomponent), C3 (complement component 3) and C4 (complement component 4). The gene expression data suggests the presence of complement pathway activators in the aged RPE/choroid. Notably, the expression level of C3 in the RPE/choroid from old animals was 9.4 fold of that in young tissue.

Ten genes involved in NK cell signaling showed increased expression in aged RPE/choroid (p<10^−6^) (Table 1; Fig. 1 B and 2 A; Fig. S1). There was increased expression of receptors and their tyrosine kinase related proteins, which mediate the activation of NK cell [28], including FCER1G (Fc fragment of IgE receptor), FCGR3A (Fc fragment of IgG receptor), DAP12 (TYRO protein tyrosine kinase binding protein) and LCK (lymphocyte-specific protein tyrosine kinase). The common effector for these receptors, SYK (spleen tyrosine kinase) and its several downstream molecules, SH3BP2 (SH3-domain binding protein 2), VAV1 (vav 1 oncogene) and RAC2 (rho family, small GTP binding protein Rac2), which are centrally involved in the transduction of signals from immune receptors [29], also showed significantly increased expression.

Interestingly, the RPE/choroid from old animals was also associated with increased expression of genes that negatively regulate inflammatory responses, notably IL-10 signaling (p<10^−5^) (Table 1; Fig. 1 B and 2 A; Fig. S2). Expression of seven members in this pathway were increased with age, including the membrane receptor IL10RA (IL-10 receptorα), CCR1 (chemokine receptor 1), FCGR2 (Fc fragment of IgG receptor) and HMOX1 (heme oxygenase 1).

### Activation of leukocyte extravasation pathway in the aged RPE/choroid

**Figure 5:**
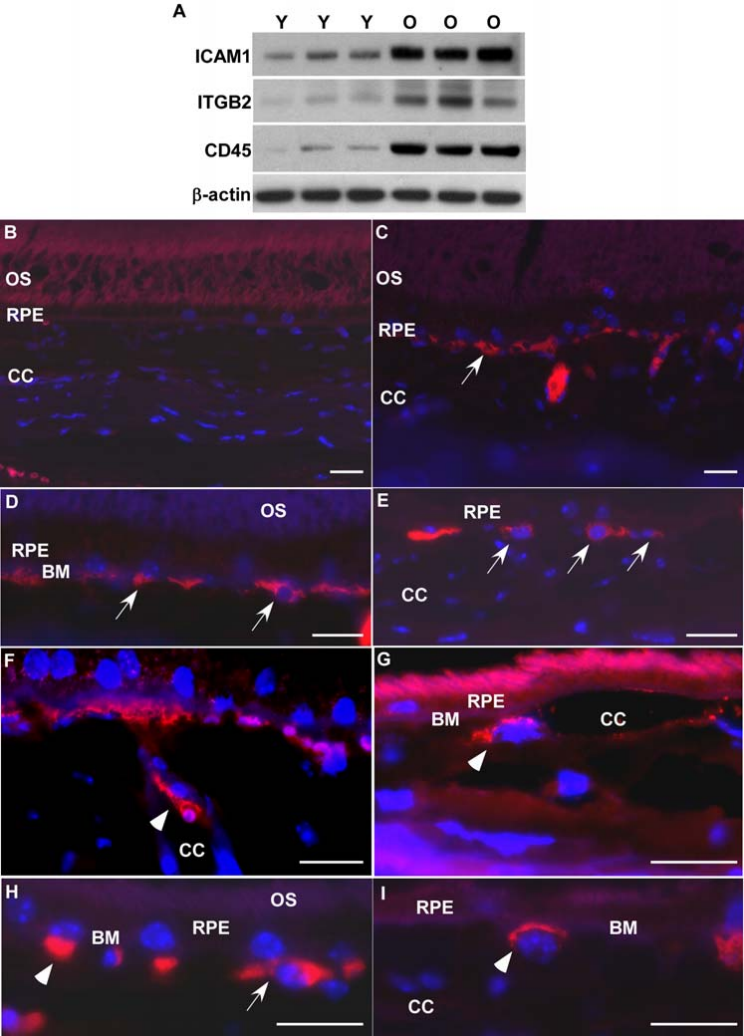
Active leukocytes recruitment in aged RPE/choroid. (A) Comparison of protein levels of ICAM1, ITGB2 and CD45 in RPE/choroid of young (Y, n = 3) and old (O, n = 3) mice by immunoblot. ICAM1, ITGB2 and CD45 are all increased in RPE/choroids from old animals. β-actin was used as a loading control. (B–I) Localization of leukocytes in young and old RPE/choroid. Leukocytes are labeled with leukocyte common antigen, CD45 (red). (B) In the young RPE/choroid, there are no leukocytes attached to Bruch's membrane or in the RPE layer. (C–I) Leukocytes in the old RPE/choroid. There are many leukocytes attached to Bruch's membrane in the RPE/choroid in old animals (C–E, arrows). Note the leukocyte that is attaching to the endothelial surface of the choroidal capillary (F, arrow heads), the leukocyte that migrated from the vessel to the local tissue (G, arrow heads), and the leukocyte passing through Bruch's membrane (H, arrow heads). The leukocyte attaching to Bruch's membrane has a lobated nucleus, indicating a polymorphonuclear leukocyte (I, arrow heads). OS, outer segment; RPE, retinal pigment epithelium; CC, choroidal capillaries; BM, Bruch's membrane. Scale bar = 20 µm.

The most significant changes in gene expression were in leukocyte extravasation pathways in the aged RPE/choroid. We also found changes in the levels of proteins that participate in extravasation of leukocytes in aged RPE/choroid. Consistent with the gene expression, immunoblots demonstrated ICAM1 and ITGB2, key molecules for the interaction between endothelial cells and leukocytes, and leukocyte common antigen CD45, the leukocyte marker, increased significantly in RPE/choroid from old animals (Fig. 5 A).

Our interpretation of the gene expression and protein profiles is that the aged RPE/choroid has become immunologically active. Based on the upregulation of the genes and proteins of the leukocyte extravasation signaling pathway, we predicted that we should find leukocytes in the RPE/choroid from normal, old animals but not in tissue from young animals. Thus, we determined the presence of leukocytes in the RPE/choroid by using CD45, which recognizes all leukocytes including granulocytes, lymphocytes and macrophages.

The immunofluorescent labeling for leukocytes showed that there were marked differences between RPE/choroid tissue from young and old animals (Fig. 5 B–I). In the young tissue, there were no leukocytes attached to Bruch's membrane or in the RPE (Fig. 5 B). In contrast, in the RPE/choroid from old animals, there were many leukocytes attached to Bruch's membrane (Fig. 5 C–E). Some leukocytes attached to the endothelial surface of the choroidal capillaries (Fig. 5 F), some leukocytes migrated from the vessels to the local tissue (Fig. 5 G), and some leukocytes were passing through Bruch's membrane (Fig. 5 H). Thus, the RPE/choroid in old animals has been invaded by leukocytes. These findings suggest an active recruitment of leukocytes from the choroidal circulation by the normal, aged RPE.

### Activation of complement pathway in the aged RPE/choroid

**Figure 6:**
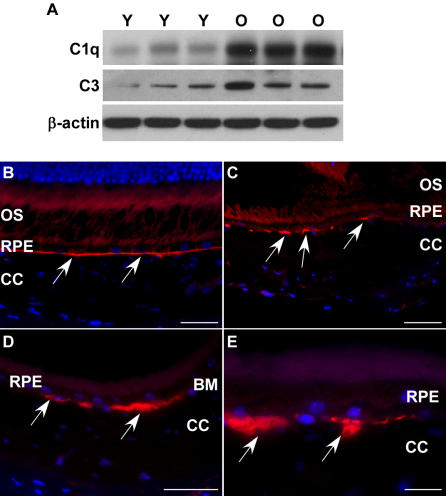
Activation of complement pathway in RPE/choroid of old animals. (A) Comparison of protein levels of C1q and C3 in RPE/choroid of young (Y, n = 3) and old (O, n = 3) mice by immunoblot. Both C1q and C3 are increased in aged RPE/choroids. β-actin was used as a loading control. (B–E) C3 deposition (red) in RPE/choroids. (B) C3 staining shows a thin and continuous line at Bruch's membrane in young animals (arrows). (C–E) In old animals, C3 deposition shows large and discontinuous clumps at Bruch's membrane (arrows). Scale bar = 50 µm (B–D) or 20 µm (E).

Based on the upregulated genes in the complement pathway in aged RPE/choroid, we determined the levels of two important activator proteins in aged RPE/choroid. C1 is the initial activator of the classical complement pathway, and C3 is the activator of the alternative pathway and the common pathway. Immunoblots showed the protein level of C1q, one of the components of C1, and C3, were increased in the aged tissue (Fig. 6 A), indicating the complement pathway was activated in the normal, aged RPE/choroid.

Immunofluorescent labeling showed there was C3 deposition on Bruch's membrane in both young and old animals, but with different deposit patterns. In young animals, C3 presented as a thin and continuous line associated with Bruch's membrane (Fig. 6 B). However, in old animals, C3 deposition showed large and discontinuous clumps on Bruch's membrane (Fig. 6 C–E), which was consistent with the increased amount of C3 in aged RPE/choroid. The discontinuous deposition of C3 may imply that the aged Bruch's membrane might be damaged and fractured. These findings suggested that the complement pathway was activated in the normal, aged RPE/choroid.

### Identifying the leukocyte attracting signal released from the aged RPE/choroid

**Figure 7:**
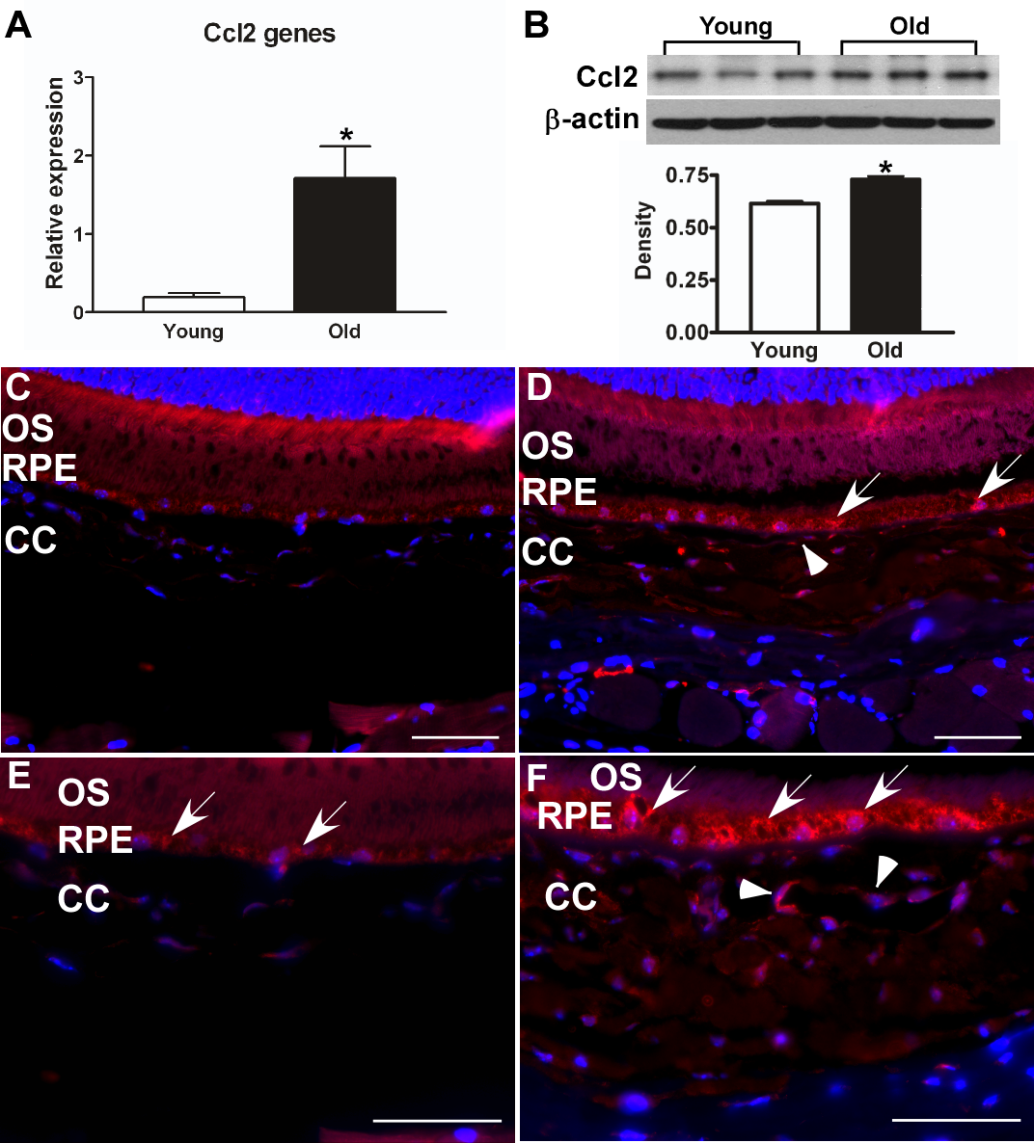
Increase of Ccl2 in RPE/choroid of old animals. (A) Comparison of Ccl2 gene expression in RPE/choroid between young and old mice. The expression level of Ccl2 is significantly increased in aged RPE/choroid (*p<0.01, n = 4). (B) Comparison of protein levels of Ccl2 in RPE/choroid of young (Y, n = 3) and old (O, n = 3) mice by immunoblot. Ccl2 is increased in aged RPE/choroids. Densitometric measurements confirm a significant increase in Ccl2 protein level in aged RPE/choroid (*p<0.01, n = 3). (C–F) Ccl2 distribution in RPE/choroid. Ccl2 is expressed in RPE cells (arrows) and endothelial cells (arrowheads). There is weak labeling for Ccl2 in the young RPE/choroid (C, E); and the amount of Ccl2 appears increased in old animals, especially in the choroid (D, F). Scale bar = 50 µm.

The above results suggested that there were signals from the aged RPE/choroid which recruited leukocytes from the circulation. Chemokine (C-C motif) ligand 2 (Ccl2) is a potent chemotactic factor which attracts multiple leukocyte populations, including monocytes, basophils, NK cells, T lymphocytes, dendritic cells and neutrophils [30]. Our microarray data showed Ccl2 was 1.6-fold increased in aged RPE/choroid (p<10^−6^). Real-time PCR confirmed that the gene expression of Ccl2 was significantly increased in aged RPE/choroid (Fig. 7 A; p<0.01, n = 4) with a higher fold change of 7.8. Immunoblot also showed that the protein level of Ccl2 was significantly increased in the aged RPE/choroid compared to tissue from young animals (Fig. 7 B; p<0.01, n = 3).

Immunofluorescent labeling showed there was some weak labeling for Ccl2 in the young RPE/choroid but Ccl2 labeling was greatly increased in the aged RPE/choroid. In the aged RPE/choroid, Ccl2 was present in RPE cells and endothelial cells (Fig. 7 C–F). Ccl2 may be one of the signals released from the normal, aged RPE to attract leukocytes from the circulation. Real-time PCR showed no significant difference for the gene expression of chemokine (C-C motif) receptor 2 (Ccr2), the primary receptor for Ccl2, between young and old (data not shown).

## Discussion

There are phenotypic changes that occur in the neural retina and in the RPE/choroid under normal aging conditions. Our findings put into context human genetic studies demonstrating gene mutations associated with AMD and animal models that exhibit AMD like changes. Our results using comprehensive microarray analyses, RT-PCR, immunoblots and immunofluorescent labeling demonstrate that there are phenotypic changes in the aged RPE/choroid that are associated with immune response effectors and pathways. The differences in the gene expression profiles comparing RPE/choroid and the adjacent neural retina from old animals are striking. We interpret our results to indicate that the RPE/choroid in the normal, old mouse has become an immunologically active tissue. This interpretation is consistent with a report on limited microarray data using aged RPE/choroid [31].

In the aged RPE/choroid, there is marked upregulation of genes and proteins that participate in leukocyte extravasation. The immunofluorescent labeling of abundant leukocytes, which may include macrophages, lymphocytes and granulocytes, attaching to Bruch's membrane in the old animals extends our gene expression and protein profiles to suggest functionality. These leukocytes may be recruited into the local tissue for removing cellular waste products or suppressing some activity in the normal aging process. The accumulation of leukocytes at Bruch's membrane and RPE suggests that there are signals from the aged RPE/choroid which recruit leukocytes from the circulation. From our data, the increased gene expression and protein production of Ccl2, but not Ccr2, in the aged RPE/choroid provides evidence in support of Ccl2 being one of those signals. This result is consistent with the findings in a recent animal model study. Ambati et al [32] report an increase in macrophages in the aged RPE/choroid of wild type animals and that Ccl2^−/−^ or Ccr2^−/−^ mice, which have a defect in macrophage recruitment, exhibit the principal hallmarks of AMD after 16 months of age. Ccl2 appears to be an important leukocyte attracting signal released from the RPE/choroid of old animals.

Genetic studies have identified mutations in CFH, the negative regulator of the complement cascade, as a gene responsible for AMD [33]–[36]. CFH inhibits the alternative complement cascade and also regulates the common pathway by inactivating C3b. Also, recent findings have demonstrated the association of AMD with two other complement genes, BF and C2, which are positive regulators of the complement cascade [17]. CFH, C3b/iC3b and BF have been colocalized in drusen and Bruch's membrane, suggesting these components of the complement pathway participate in drusen formation [17], [37]. A recent study showed an increased membrane attack complex (C5b-9) in Bruch's membrane of normal, aged human retina [38]. In addition, knockout of CFH in mice causes AMD-like changes when the animals age [39]. Our findings in the normal aged RPE/choroid fit well with these previous observations. For example, our microarray data have a consistent finding of increased gene expression of complement pathway genes, especially C3. The protein levels of complement cascade activators, C1q and C3, are increased in the aged RPE/choroid. Increased C3 protein is present as clumps of deposits on Bruch's membrane in old eyes. All of these results suggest that the complement cascade is activated in the RPE/choroid of old animals. We extrapolate our findings to predict that an error in the complement pathway will not cause AMD until the age-related changes leading to increased immunological activity appear in the elderly adult.

The physiological function of IL-10 signaling is to regulate macrophages and generate anti-inflammatory responses [40]. Several studies have demonstrated that interleukins (IL) including IL-6, -8, and -10 are associated with AMD [41]–[43]. Our data show several genes in IL-10 signaling are upregulated. Whether the activation of IL-10 signaling is triggered directly by the aged RPE/choroid or is a feedback response from the increased immunological activity of the aged RPE/choroid is not known. However, the increased activity of both positive and negative pathway regulation in the RPE/choroid of old animals suggests that a balanced mechanism is crucial for this tissue to age properly.

We hypothesize that the aged RPE/choroid has become intensely immunologically active and synthesizes proteins that attract leukocytes and activate the complement pathway. Activation of these pathways, which are part of normal aging in the RPE/choroid, requires extremely tight control by an intricate series of activating and regulatory proteins. To the extent that these age-related activities occur in the human RPE/choroid, any dysfunction of this normal aging process, e.g. through defects in BF, C2 or CFH, may predispose the aged RPE/choroid to AMD. In humans, small differences in genomic DNA among individuals can produce a spectrum of influences on immunological activities. Polymorphisms, mutations or single nucleotide polymorphisms (SNPs), which lead to changes of the normal immunological activities in the aged RPE/choroid, have the potential of being disease causing factors or susceptibility factors for the development of AMD. We hypothesize that phenotypic changes in the aged, human RPE/choroid provides the background for an error in regulation of immunological activity to cause AMD to appear in an elderly individual.

## Materials and Methods

### Mice

All protocols were in compliance with NIH guidelines and approved by the Center for Comparative Medicine Committee at Northwestern University. C57BL/6 male mice, 4 mos (young) and 26 mos (old) of age (NIA, Bethesda, MD) were used. Animals were housed under standard conditions.

### RNA Isolation

Mice neural retina and RPE/choroid were separated and placed in RNA lysis buffer and RNA was extracted by using the RNeasy Minikit (Qiagen). There were 12 neural retina and 8 RPE/choroid samples for microarray. Integrity and concentration of RNA was evaluated using an Agilent 2100 Bioanalyzer (Agilent Technologies).

### Microarray analysis

The labeling and hybridization of the cRNA was performed at the Functional Genomics Facility at The University of Chicago. The cRNA were prepared following the Affymetrix GeneChip Expression Analysis Manual. 12 µg of labeled cRNA were hybridized to Affymetrix Mouse 430 2.0 arrays for 16 hrs at 45°C and 60 rpm in an Affymetrix Hybridization Oven 640. The arrays were washed and stained with streptavidin phycoerythrin in Affymetrix Fluidics Station 450 following Affymetrix GeneChip protocol and then scanned using an Affymetrix GeneChip Scanner 3000.

### Microarray data statistical analysis

The acquisition and initial quantification of array images were performed using the GCOS (GeneChip Operating Software, Affymetrix). The*.CEL files were obtained from GCOS.

We used three different methods to identify the differentially expressed genes. We first used packages in Bioconductor [44] for data analysis. The data were preprocessed and normalized using the RMA method in affy package [45]. To reduce false positives, we removed the probe-sets with all samples as “Absent” in the data analysis. The probe-set “Present”/“Absent” calls were estimated using the Wilcoxon signed rank-based algorithm [46] implemented in affy package. Then we identified the differentially expressed gene list by the empirical Bayes shrinkage moderated t-statistics in Limma Bioconductor package [47]. We considered the genes with fold-change >2.0 and false discovery rate (FDR) [48] adjusted p<0.01 as significantly differentially expressed. The second method we used was Dchip [49]. Invariant set normalization was performed using Dchip2006 and perfect match-only model was used for generating gene signal intensities. Genes that met the criteria fold change >2 and statistical difference p<0.05 simultaneously were considered as significant changes. The third method was SAM [50]. Microarray analysis was done with the SAM module within BRB-Array tools (Version 3.4, NCI). Genes were first filtered in BRB array tools to exclude genes where less than 20% of the expression data had a 1.5-fold change in either direction and where the missing or filtered out data exceeded 50%. After this filtering, 1451 genes were submitted for SAM analysis. The criterion for selection was a 1% FDR with 90% percentile confidence.

After removal of the repeated and unannotated genes, the identified differentially expressed genes were further analyzed for global functional analyses, network analyses and canonical pathway analyses using Ingenuity Pathway Analysis (Ingenuity® Systems, www.ingenuity.com). A differentially expressed gene list containing gene identifiers and corresponding fold changes was uploaded as an Excel spreadsheet into the software. Each gene identifier was mapped to its corresponding gene object in the Ingenuity Pathways Knowledge Base. We also used GOstats Bioconductor package[44] for the Gene Ontology (GO) analysis.

### Real-time RT-PCR

100 ng of total RNA (400 ng of total RNA for Ccl2 gene) from each sample was reverse transcribed into cDNA using iScript cDNA Synthesis Kit (Bio-Rad) and used as a template for real-time PCR reactions. Specific primers (Table 2) were synthesized by Integrated DNA Technologies. The RT-PCR reactions were performed using iCycler (Bio-Rad). All samples were tested in triplicate PCR reactions and the mean of the reactions was used for calculating the expression levels. Expression levels were normalized to 18S mRNA and analyzed by a standard two-tailed t-test using GraphPad Prism (GraphPad Software, Inc).

**Table 2:** Gene-specific primers used in the study.

Gene	Forward primer	Reverse primer
**ICAM1**	**tggtgatgctcaggtatcca**	**ggtccactctcgagctcatc**
**ITGB2**	**tctgcagtaatggagcatcg**	**tcctggatacactcggaagc**
**C3**	**tcagatggctgaagatgctg**	**ctgttggtgtcatgccaatc**
**C1QC**	**gttcaacagcaagcaggtca**	**accagagaagacgctgttgg**
**FCER1G**	**cttgctccttttggtggaac**	**ctggctatagctgcctttcg**
**SH3BP2**	**gggaccattgcctctcaata**	**accacacattagggctgctc**
**SYK**	**gatgggctctacctgctacg**	**atggcgtaggtgccattaag**
**CCR1**	**accagttctatatctccatc**	**ctcattctcttcttagacct**
**IL1RN**	**acagtcacctaatctctctc**	**aaggtcttctggttagtatc**
**BCL2A1**	**tacaggtacccgcctttgag**	**ttccacgtgaaagtcatcca**
**CD45**	**atcatcgccagcatctatcc**	**ctggacggacacagttagca**
**Ccl2**	**tactcattcaccagcaaga**	**gagcttggtgacaaaaacta**
**Ccr2**	**gaaaggtttctgtcaggtta**	**caggaagaggttgagagata**
**18S**	**gtaacccgttgaaccccatt**	**ccatccaatcggtagtagcg**

### Immunoblot analysis

The RPE/choroid was lysed in extraction buffer containing 50 mM Tris-HCl (pH 8.0), 150 mM NaCl, 1 mM EDTA plus protease inhibitors and phosphatase inhibitors (Pierce) and sonicated for 5 sec before protein concentration determination by the Bradford colorimetric assay. 20 µg proteins (40 µg of RPE/choroid protein for Ccl2 detection) were loaded onto 4–12% SDS-PAGE gels and electrotransferred on polyvinylidene fluoride (PVDF) membranes (Amersham). The membrane was blocked for 1 hr at room temperature in blocking solution containing 5% nonfat milk and 0.05% Tween 20 in Tris-buffered saline (TBS) (25 mM Tris-HCl and 150 mM NaCl) and then incubated with primary antibodies in blocking solution at 4°C overnight. The following primary antibodies were used: goat anti-ICAM1 (1∶200; Santa Cruz), rat anti-ITGB2 (1∶100; Santa Cruz), mouse anti-CD45 (1∶1000; BD Biosciences), rat anti-C1q (1∶100, HyCult biotechnology), rabbit anti-C3 (1∶100; abcam), rabbit anti-Ccl2 (1∶1000; abcam) and mouse anti-β-actin (1∶20,000; Sigma).The membrane was rinsed with 0.05% Tween 20 in TBS and incubated with peroxidase-conjugated donkey anti-goat IgG (1∶15,000; Santa Cruz), goat anti-rat IgG (1∶10,000), goat anti-mouse IgG (1∶10,000) or donkey anti-rabbit IgG (1∶15,000) for 1 hr at room temperature. Finally, the blots were developed by enhanced chemiluminescence (ECL) (Pierce) on Hyperfilm (Amersham). The immunoblots for Ccl2 were scanned and relative band density was determined using ImageJ (National Institutes of Health, Bethesda, MD). The densities were normalized to β-actin and analyzed by a standard two-tailed t-test using GraphPad Prism.

### Immunofluorescent labeling

Eyes were fixed in 4% paraformaldehyde in PBS for 4 hrs and embedded in paraffin. 5 µm thick sections were de-waxed and rehydrated. For antigen retrieval, the sections were heated in 10 mM sodium citrate buffer (pH 6.0) at a sub boiling temperature for 10 min followed by cooling for 30 min. The tissue sections were incubated with mouse anti-CD45 (1∶200), rabbit anti-C3 (1∶100) or rabbit anti-Ccl2 (1∶100; Chemicon) diluted in 5% BSA in PBS for overnight at 4°C. After several washes, tissue sections were incubated with the secondary antibody, Alexa Fluor 568 goat anti-mouse IgG or donkey anti-rabbit IgG (1∶800; Molecular Probes) for 1 hr at room temperature. After washing with PBS, slides were mounted with Vectorshield containing DAPI (Vector Laboratory) and observed using fluorescence microscopy.

## Supporting Information

Table S1 Genes that are differentially expressed with age in the RPE/choroid
(0.44 MB DOC)

Figure S1 Pathway diagram showing the molecules involved in NK cell signaling and their interaction. The diagram was modified from Ingenuity Pathway Analysis (Ingenuity® Systems).
(3.73 MB TIF)

Figure S2 Pathway diagram showing the molecules involved in IL-10 signaling and their interaction. The diagram was modified from Ingenuity Pathway Analysis (Ingenuity® Systems).
(9.74 MB TIF)
