# Supplementary material for: The Aged Retinal Pigment Epithelium/Choroid: A Potential Substratum for the Pathogenesis of Age-Related Macular Degeneration
Source: PLoS One. 2008 Jun 4;3(6):e2339. doi: 10.1371/journal.pone.0002339 (PMC2394659; doi:10.1371/journal.pone.0002339)
Supplement: Table S1 — Genes that are differentially expressed with age in the RPE/choroid (0.44 MB DOC) [file pone.0002339.s001.doc]

| Table S1. Genes that are differentially expressed with age in the RPE/choroid | | | | |
| --- | --- | --- | --- | --- |
| Entrez Id | Gene Symbol | Gene Name | Fold | p value |
| **Genes upregulated** | |  |  |  |
| 18405 | Orm1 | orosomucoid 1 | 28.90 | 5.04E-05 |
| 16061 | Igh-VJ558 | immunoglobulin heavy chain (J558 family) | 19.88 | 0.00010571 |
| 16114 | Igk-V28 | immunoglobulin kappa chain variable 28 (V28) | 14.87 | 1.85E-05 |
| 16069 | Igj | immunoglobulin joining chain | 13.33 | 7.69E-05 |
| 17392 | Mmp3 | matrix metallopeptidase 3 | 12.22 | 1.85E-05 |
| 228801 | U46068 | cDNA sequence U46068 | 11.68 | 9.70E-06 |
| 12266 | C3 | complement component 3 | 9.38 | 3.47E-05 |
| 17386 | Mmp13 | matrix metallopeptidase 13 | 9.11 | 0.00031966 |
| 246746 | Cd300lf | CD300 antigen like family member F | 7.62 | 0.00011414 |
| 16819 | Lcn2 | lipocalin 2 | 7.13 | 0.0063853 |
| 21923 | Tnc | tenascin C | 6.68 | 1.24E-05 |
| 16197 | Il7r | interleukin 7 receptor | 6.61 | 6.76E-05 |
| 12182 | Bst1 | bone marrow stromal cell antigen 1 | 6.50 | 0.00031828 |
| 11501 | Adam8 | a disintegrin and metallopeptidase domain 8 | 5.59 | 0.00027218 |
| 329002 | Zfp236 | zinc finger protein 236 | 5.55 | 0.00014541 |
| 20717 | Serpina3m | serine (or cysteine) peptidase inhibitor, clade A, member 3M | 5.48 | 0.00014637 |
| 60361 | Ms4a4b | membrane-spanning 4-domains, subfamily A, member 4B | 5.39 | 0.00013749 |
| 19218 | Ptger3 | prostaglandin E receptor 3 (subtype EP3) | 5.25 | 0.00014541 |
| 13371 | Dio2 | deiodinase, iodothyronine, type II | 5.05 | 3.98E-05 |
| 15559 | Htr2b | 5-hydroxytryptamine (serotonin) receptor 2B | 4.94 | 1.45E-06 |
| 17329 | Cxcl9 | chemokine (C-X-C motif) ligand 9 | 4.90 | 0.0033069 |
| 16411 | Itgax | integrin alpha X | 4.88 | 6.07E-05 |
| 12984 | Csf2rb2 | colony stimulating factor 2 receptor, beta 2, low-affinity | 4.78 | 3.30E-05 |
| 12983 | Csf2rb1 | colony stimulating factor 2 receptor, beta 1, low-affinity | 4.64 | 6.61E-05 |
| 12032 | Bcan | brevican | 4.53 | 0.0001661 |
| 78754 | Galntl2 | polypeptide N-acetylgalactosaminyltransferase-like 2 | 4.49 | 0.00054402 |
| 71738 | Mamdc2 | MAM domain containing 2 | 4.48 | 0.00016608 |
| 18722 | Pira1 | paired-Ig-like receptor A1 | 4.40 | 0.00015333 |
| 20307 | Ccl8 | chemokine (C-C motif) ligand 8 | 4.36 | 0.0012239 |
| 20210 | Saa3 | serum amyloid A 3 | 4.30 | 0.00014541 |
| 13837 | Epha3 | Eph receptor A3 | 4.28 | 1.85E-05 |
| 18729 | Pira6 | paired-Ig-like receptor A6 | 4.26 | 0.00020964 |
| 56619 | Clec4e | C-type lectin domain family 4, member e | 4.22 | 0.0010814 |
| 12502 | Cd3g | CD3 antigen, gamma polypeptide | 4.10 | 0.00020678 |
| 80901 | Cxcr6 | chemokine (C-X-C motif) receptor 6 | 3.96 | 0.00088513 |
| 50490 | Nox4 | NADPH oxidase 4 | 3.95 | 1.85E-05 |
| 14268 | Fn1 | fibronectin 1 | 3.94 | 2.62E-05 |
| 109648 | Npy | neuropeptide Y | 3.89 | 0.00037129 |
| 58860 | Adamdec1 | ADAM-like, decysin 1 | 3.83 | 0.00016689 |
| 110595 | Timp4 | tissue inhibitor of metalloproteinase 4 | 3.82 | 3.32E-05 |
| 239849 | Cd200r4 | Cd200 receptor 4 | 3.77 | 1.52E-05 |
| 11770 | Fabp4 | fatty acid binding protein 4, adipocyte | 3.76 | 0.00019117 |
| 11433 | Acp5 | acid phosphatase 5, tartrate resistant | 3.71 | 0.00010934 |
| 242341 | Atp6v0d2 | ATPase, H+ transporting, lysosomal V0 subunit D2 | 3.66 | 5.35E-05 |
| 17304 | Mfge8 | milk fat globule-EGF factor 8 protein | 3.65 | 5.23E-05 |
| 320832 | Sirpb1 | signal-regulatory protein beta 1 | 3.63 | 0.00022051 |
| 20311 | Cxcl5 | chemokine (C-X-C motif) ligand 5 | 3.55 | 0.00020579 |
| 16165 | Il13ra2 | interleukin 13 receptor, alpha 2 | 3.55 | 0.00046733 |
| 52855 | Lair1 | leukocyte-associated Ig-like receptor 1 | 3.53 | 0.00030431 |
| 68279 | Mcoln2 | mucolipin 2 | 3.48 | 6.88E-05 |
| 12583 | Cdo1 | cysteine dioxygenase 1, cytosolic | 3.44 | 0.00024232 |
| 16196 | Il7 | interleukin 7 | 3.42 | 6.61E-05 |
| 12873 | Cpa3 | carboxypeptidase A3, mast cell | 3.40 | 0.0028231 |
| 16409 | Itgam | integrin alpha M | 3.40 | 0.00017275 |
| 16181 | Il1rn | interleukin 1 receptor antagonist | 3.36 | 6.61E-05 |
| 14229 | Fkbp5 | FK506 binding protein 5 | 3.36 | 0.0025352 |
| 74152 | 1300002K09Rik | RIKEN cDNA 1300002K09 gene | 3.31 | 5.39E-05 |
| 14130 | Fcgr2b | Fc receptor, IgG, low affinity IIb | 3.31 | 5.79E-05 |
| 17474 | Clec4d | C-type lectin domain family 4, member d | 3.28 | 0.00027162 |
| 16634 | Klra3 | killer cell lectin-like receptor, subfamily A, member 3 | 3.26 | 0.00010291 |
| 68774 | Ms4a6d | membrane-spanning 4-domains, subfamily A, member 6D | 3.25 | 5.68E-05 |
| 16414 | Itgb2 | integrin beta 2 | 3.23 | 8.51E-05 |
| 23845 | Clec5a | C-type lectin domain family 5, member a | 3.22 | 7.69E-05 |
| 12323 | Camk2b | calcium/calmodulin-dependent protein kinase II, beta | 3.21 | 0.0024742 |
| 12523 | Cd84 | CD84 antigen | 3.21 | 5.79E-05 |
| 65221 | Slc15a3 | solute carrier family 15, member 3 | 3.20 | 0.00015333 |
| 226691 | AI607873 | expressed sequence AI607873 | 3.20 | 0.00022235 |
| 19200 | Pstpip1 | proline-serine-threonine phosphatase-interacting protein 1 | 3.15 | 0.00016248 |
| 18733 | Lilrb3 | leukocyte immunoglobulin-like receptor, subfamily B, member 3 | 3.15 | 0.00011505 |
| 69073 | 1810019J16Rik | RIKEN cDNA 1810019J16 gene | 3.12 | 0.00073191 |
| 21426 | Tcfec | transcription factor EC | 3.12 | 5.04E-05 |
| 14728 | Lilrb4 | leukocyte immunoglobulin-like receptor, subfamily B, member 4 | 3.11 | 5.29E-05 |
| 13038 | Ctsk | cathepsin K | 3.09 | 0.00010236 |
| 56193 | Plek | pleckstrin | 3.07 | 3.68E-05 |
| 68509 | 1110018H23Rik | RIKEN cDNA 1110018H23 gene | 3.03 | 0.00012293 |
| 16658 | Mafb | v-maf musculoaponeurotic fibrosarcoma oncogene family | 3.01 | 5.35E-05 |
| 70789 | Kynu | kynureninase (L-kynurenine hydrolase) | 3.01 | 0.00011001 |
| 14421 | B4galnt1 | beta-1,4-N-acetyl-galactosaminyl transferase 1 | 3.00 | 0.00043132 |
| 76633 | 1700112E06Rik | RIKEN cDNA 1700112E06 gene | 2.98 | 1.52E-05 |
| 320207 | Pik3r5 | phosphoinositide-3-kinase, regulatory subunit 5, p101 | 2.96 | 0.00031618 |
| 27052 | Aoah | acyloxyacyl hydrolase | 2.95 | 5.79E-05 |
| 103814 | AI662270 | expressed sequence AI662270 | 2.94 | 5.23E-05 |
| 12767 | Cxcr4 | chemokine (C-X-C motif) receptor 4 | 2.94 | 0.00024232 |
| 57781 | Cd200r1 | CD200 receptor 1 | 2.93 | 0.00012293 |
| 13058 | Cybb | cytochrome b-245, beta polypeptide | 2.93 | 3.30E-05 |
| 67702 | Rnf149 | ring finger protein 149 | 2.91 | 6.61E-05 |
| 433470 | AA467197 | expressed sequence AA467197 | 2.90 | 0.0084707 |
| 12262 | C1qc | complement component 1, q subcomponent, C chain | 2.89 | 5.84E-05 |
| 56318 | Acpp | acid phosphatase, prostate | 2.89 | 0.00023355 |
| 19716 | Bex1 | brain expressed gene 1 | 2.88 | 0.0010785 |
| 279572 | Tlr13 | toll-like receptor 13 | 2.88 | 0.00011347 |
| 107321 | Lpxn | leupaxin | 2.86 | 0.00054402 |
| 12768 | Ccr1 | chemokine (C-C motif) receptor 1 | 2.86 | 0.00083896 |
| 73690 | Glipr1 | GLI pathogenesis-related 1 (glioma) | 2.85 | 0.00011814 |
| 12493 | Cd37 | CD37 antigen | 2.84 | 5.04E-05 |
| 12514 | Cd68 | CD68 antigen | 2.83 | 5.79E-05 |
| 80719 | Igsf6 | immunoglobulin superfamily, member 6 | 2.81 | 0.00020253 |
| 27083 | Xlr4b | X-linked lymphocyte-regulated 4B | 2.80 | 0.006107 |
| 94284 | Ugt1a6a | UDP glucuronosyltransferase 1 family, polypeptide A6A | 2.79 | 6.07E-05 |
| 20491 | Sla | src-like adaptor | 2.79 | 0.00016067 |
| 102538 | AI467657 | expressed sequence AI467657 | 2.78 | 0.0059682 |
| 54673 | Sh3glb1 | SH3-domain GRB2-like B1 (endophilin) | 2.77 | 0.002181 |
| 12044 | Bcl2a1a | B-cell leukemia/lymphoma 2 related protein A1a | 2.75 | 5.39E-05 |
| 20292 | Ccl11 | small chemokine (C-C motif) ligand 11 | 2.74 | 0.0027212 |
| 20716 | Serpina3n | serine (or cysteine) peptidase inhibitor, clade A, member 3N | 2.74 | 0.0029059 |
| 72310 | Nkg7 | natural killer cell group 7 sequence | 2.73 | 0.0027432 |
| 76942 | Lypd5 | Ly6/Plaur domain containing 5 | 2.73 | 0.00029546 |
| 171285 | Havcr2 | hepatitis A virus cellular receptor 2 | 2.72 | 6.49E-05 |
| 16792 | Laptm5 | lysosomal-associated protein transmembrane 5 | 2.72 | 5.68E-05 |
| 83433 | Trem2 | triggering receptor expressed on myeloid cells 2 | 2.71 | 6.76E-05 |
| 246256 | Fcgr3a | Fc fragment of IgG, low affinity IIIa, receptor | 2.71 | 0.0014469 |
| 16416 | Itgb3 | integrin beta 3 | 2.71 | 0.00099071 |
| 12845 | Comp | cartilage oligomeric matrix protein | 2.70 | 0.00048953 |
| 76612 | Lrrc27 | leucine rich repeat containing 27 | 2.70 | 0.00010106 |
| 207839 | Galnt6 | polypeptide N-acetylgalactosaminyltransferase 6 | 2.69 | 0.00027115 |
| 98267 | Stk17b | serine/threonine kinase 17b (apoptosis-inducing) | 2.68 | 0.0018039 |
| 12363 | Casp4 | caspase 4, apoptosis-related cysteine peptidase | 2.65 | 0.00011347 |
| 22324 | Vav1 | vav 1 oncogene | 2.64 | 5.35E-05 |
| 114564 | Csprs | component of Sp100-rs | 2.63 | 0.00020579 |
| 11690 | Alox5ap | arachidonate 5-lipoxygenase activating protein | 2.62 | 0.0010273 |
| 16154 | Il10ra | interleukin 10 receptor, alpha | 2.62 | 6.15E-05 |
| 22436 | Xdh | xanthine dehydrogenase | 2.62 | 0.00027204 |
| 24055 | Sh3bp2 | SH3-domain binding protein 2 | 2.62 | 0.00024365 |
| 20538 | Slc6a2 | solute carrier family 6, member 2 | 2.61 | 0.0012531 |
| 108101 | BC032204 | cDNA sequence BC032204 | 2.60 | 0.00012293 |
| 14125 | Fcer1a | Fc receptor, IgE, high affinity I, alpha polypeptide | 2.60 | 0.0064 |
| 17228 | Cma1 | chymase 1, mast cell | 2.59 | 0.0064766 |
| 16408 | Itgal | integrin alpha L | 2.59 | 0.00041451 |
| 17972 | Ncf4 | neutrophil cytosolic factor 4 | 2.59 | 0.00037003 |
| 12526 | Cd8b1 | CD8 antigen, beta chain 1 | 2.59 | 0.00067704 |
| 16182 | Il18r1 | interleukin 18 receptor 1 | 2.58 | 0.0041675 |
| 654812 | Angptl7 | angiopoietin-like 7 | 2.58 | 0.0015687 |
| 67133 | Gp2 | glycoprotein 2 (zymogen granule membrane) | 2.57 | 0.0017711 |
| 12260 | C1qb | complement component 1, q subcomponent, beta polypeptide | 2.57 | 5.24E-05 |
| 20288 | Msr1 | macrophage scavenger receptor 1 | 2.56 | 0.00012293 |
| 72054 | Cyp4f18 | cytochrome P450, family 4, subfamily f, polypeptide 18 | 2.55 | 0.0017922 |
| 170743 | Tlr7 | toll-like receptor 7 | 2.55 | 0.00041463 |
| 23833 | Cd52 | CD52 antigen | 2.55 | 0.00019117 |
| 12362 | Casp1 | caspase 1 | 2.55 | 8.49E-05 |
| 19264 | Ptprc | protein tyrosine phosphatase, receptor type, C | 2.53 | 6.75E-05 |
| 22329 | Vcam1 | vascular cell adhesion molecule 1 | 2.53 | 5.04E-05 |
| 12508 | Cd53 | CD53 antigen | 2.53 | 0.00014637 |
| 56264 | Cpxm1 | carboxypeptidase X 1 (M14 family) | 2.52 | 7.45E-05 |
| 17969 | Ncf1 | neutrophil cytosolic factor 1 | 2.52 | 6.61E-05 |
| 14131 | Fcgr3 | Fc receptor, IgG, low affinity III | 2.52 | 0.00024334 |
| 16541 | Napsa | napsin A aspartic peptidase | 2.51 | 0.00038868 |
| 58801 | Pmaip1 | phorbol-12-myristate-13-acetate-induced protein 1 | 2.50 | 0.0017455 |
| 57914 | Tpte2 | transmembrane phosphoinositide 3-phosphatase | 2.49 | 8.60E-05 |
|  |  | and tensin homolog 2 |  |  |
| 50909 | C1r | complement component 1, r subcomponent | 2.48 | 9.70E-06 |
| 140497 | Cd300d | Cd300D antigen | 2.47 | 0.00016834 |
| 20715 | Serpina3g | serine (or cysteine) peptidase inhibitor, clade A, member 3G | 2.47 | 0.0079683 |
| 15951 | Ifi204 | interferon activated gene 204 | 2.46 | 0.00033183 |
| 96875 | Prg4 | proteoglycan 4 (megakaryocyte stimulating factor) | 2.46 | 0.0036119 |
| 17076 | Ly75 | lymphocyte antigen 75 | 2.46 | 0.00041896 |
| 12399 | Runx3 | runt related transcription factor 3 | 2.46 | 0.0053179 |
| 17951 | Birc1e | baculoviral IAP repeat-containing 1e | 2.45 | 5.35E-05 |
| 20556 | Slfn2 | schlafen 2 | 2.44 | 0.0005073 |
| 12393 | Runx2 | runt related transcription factor 2 | 2.44 | 0.00026878 |
| 16365 | Irg1 | immunoresponsive gene 1 | 2.44 | 0.0049298 |
| 20963 | Syk | spleen tyrosine kinase | 2.43 | 0.00012293 |
| 107350 | AW112010 | expressed sequence AW112010 | 2.43 | 0.0027682 |
| 58223 | Mmp19 | matrix metallopeptidase 19 | 2.43 | 0.0034653 |
| 14127 | Fcer1g | Fc receptor, IgE, high affinity I, gamma polypeptide | 2.42 | 3.30E-05 |
| 18793 | Plaur | plasminogen activator, urokinase receptor | 2.42 | 0.0055312 |
| 12816 | Col12a1 | procollagen, type XII, alpha 1 | 2.42 | 0.00020784 |
| 15019 | H2-Q8 | histocompatibility 2, Q region locus 8 | 2.42 | 0.0070397 |
| 20304 | Ccl5 | chemokine (C-C motif) ligand 5 | 2.41 | 0.0014771 |
| 68655 | Fndc1 | fibronectin type III domain containing 1 | 2.41 | 0.00010112 |
| 20198 | S100a4 | S100 calcium binding protein A4 | 2.41 | 0.00024142 |
| 56644 | Clec7a | C-type lectin domain family 7, member a | 2.41 | 6.99E-05 |
| 75750 | Slc10a6 | solute carrier family 10, member 6 | 2.40 | 0.0089601 |
| 15368 | Hmox1 | heme oxygenase (decycling) 1 | 2.40 | 0.00071255 |
| 66102 | Cxcl16 | chemokine (C-X-C motif) ligand 16 | 2.40 | 9.38E-05 |
| 71712 | 1200002N14Rik | RIKEN cDNA 1200002N14 gene | 2.39 | 5.68E-05 |
| 102084 | AI451557 | expressed sequence AI451557 | 2.39 | 0.0062465 |
| 16997 | Ltbp2 | latent transforming growth factor beta binding protein 2 | 2.38 | 0.00048066 |
| 12654 | Chi3l1 | chitinase 3-like 1 | 2.38 | 1.85E-05 |
| 75345 | Slamf7 | SLAM family member 7 | 2.38 | 0.0014299 |
| 12500 | Cd3d | CD3 antigen, delta polypeptide | 2.38 | 0.00089799 |
| 19354 | Rac2 | RAS-related C3 botulinum substrate 2 | 2.37 | 0.00020253 |
| 50908 | C1s | complement component 1, s subcomponent | 2.37 | 5.24E-05 |
| 15957 | Ifit1 | interferon-induced protein with tetratricopeptide repeats 1 | 2.35 | 0.0025748 |
| 14428 | Galr2 | galanin receptor 2 | 2.35 | 0.00078584 |
| 12268 | C4b | complement component 4B (Childo blood group) | 2.34 | 6.49E-05 |
| 69774 | Ms4a6b | membrane-spanning 4-domains, subfamily A, member 6B | 2.34 | 5.24E-05 |
| 319269 | A130040M12Rik | RIKEN cDNA A130040M12 gene | 2.34 | 0.0015916 |
| 14605 | Tsc22d3 | TSC22 domain family 3 | 2.34 | 0.0016467 |
| 235505 | Cd109 | CD109 antigen | 2.34 | 6.76E-05 |
| 12483 | Cd22 | CD22 antigen | 2.33 | 7.20E-05 |
| 13197 | Gadd45a | growth arrest and DNA-damage-inducible 45 alpha | 2.33 | 0.00049597 |
| 101757 | AU020206 | expressed sequence AU020206 | 2.32 | 0.00090858 |
| 212032 | Hk3 | hexokinase 3 | 2.32 | 0.00063398 |
| 622976 | EG622976 | predicted gene, EG622976 | 2.32 | 0.00027115 |
| 12332 | Capg | capping protein (actin filament), gelsolin-like | 2.32 | 5.39E-05 |
| 22177 | Tyrobp | TYRO protein tyrosine kinase binding protein | 2.32 | 3.30E-05 |
| 170768 | Pfkfb3 | 6-phosphofructo-2-kinase/fructose-2,6-biphosphatase 3 | 2.31 | 0.00032386 |
| 20375 | Sfpi1 | SFFV proviral integration 1 | 2.31 | 0.00029959 |
| 13057 | Cyba | cytochrome b-245, alpha polypeptide | 2.30 | 5.79E-05 |
| 12259 | C1qa | complement component 1, q subcomponent, alpha polypeptide | 2.30 | 6.61E-05 |
| 21857 | Timp1 | tissue inhibitor of metalloproteinase 1 | 2.29 | 0.00039729 |
| 17948 | Birc1b | baculoviral IAP repeat-containing 1b | 2.29 | 0.0002223 |
| 17476 | Mpeg1 | macrophage expressed gene 1 | 2.28 | 7.20E-05 |
| 74499 | Sost | sclerostin | 2.28 | 0.00049977 |
| 217066 | BC099439 | cDNA sequence BC099439 | 2.27 | 0.0054096 |
| 24099 | Tnfsf13b | tumor necrosis factor (ligand) superfamily, member 13b | 2.26 | 0.00052288 |
| 20378 | Frzb | frizzled-related protein | 2.26 | 5.04E-05 |
| 269846 | Tcrb-V13 | T-cell receptor beta, variable 13 | 2.26 | 0.00031873 |
| 83924 | Gpr137b | G protein-coupled receptor 137B | 2.25 | 4.52E-05 |
| 13479 | Dpep1 | dipeptidase 1 (renal) | 2.25 | 0.0099753 |
| 13030 | Ctsb | cathepsin B | 2.24 | 0.00010202 |
| 58916 | Myot | myotilin | 2.24 | 0.00097856 |
| 14945 | Gzmk | granzyme K | 2.23 | 0.0061504 |
| 100340 | Smpdl3b | sphingomyelin phosphodiesterase, acid-like 3B | 2.23 | 0.00049775 |
| 12554 | Cdh13 | cadherin 13 | 2.23 | 5.39E-05 |
| 73723 | Sh3bgrl3 | SH3 domain binding glutamic acid-rich protein-like 3 | 2.21 | 0.00013387 |
| 15040 | H2-T23 | histocompatibility 2, T region locus 23 | 2.21 | 0.00028328 |
| 110067 | Tcrg | T-cell receptor gamma chain | 2.19 | 0.0012613 |
| 12835 | Col6a3 | procollagen, type VI, alpha 3 | 2.18 | 0.00041463 |
| 64214 | Rgs18 | regulator of G-protein signaling 18 | 2.18 | 0.00093588 |
| 19141 | Lgmn | legumain | 2.18 | 0.00029591 |
| 226841 | Vash2 | vasohibin 2 | 2.17 | 0.00076373 |
| 106267 | AI844685 | expressed sequence AI844685 | 2.16 | 0.001624 |
| 14962 | Cfb | complement factor B | 2.16 | 3.73E-05 |
| 16818 | Lck | lymphocyte protein tyrosine kinase | 2.16 | 0.00045097 |
| 11746 | Anxa4 | annexin A4 | 2.15 | 6.61E-05 |
| 18807 | Pld3 | phospholipase D family, member 3 | 2.15 | 7.11E-05 |
| 11605 | Gla | galactosidase, alpha | 2.15 | 0.00027115 |
| 226652 | Arhgap30 | Rho GTPase activating protein 30 | 2.15 | 6.61E-05 |
| 382643 | AI450948 | expressed sequence AI450948 | 2.14 | 6.61E-05 |
| 15894 | Icam1 | intercellular adhesion molecule | 2.14 | 6.26E-05 |
| 16952 | Anxa1 | annexin A1 | 2.14 | 9.38E-05 |
| 19267 | Ptpre | protein tyrosine phosphatase, receptor type, E | 2.14 | 5.06E-05 |
| 329679 | D630023B12Rik | RIKEN cDNA D630023B12 gene | 2.14 | 0.00066745 |
| 20319 | Sfrp2 | secreted frizzled-related protein 2 | 2.14 | 0.0028858 |
| 16068 | Il18bp | interleukin 18 binding protein | 2.14 | 0.0027754 |
| 53415 | Htatip2 | HIV-1 tat interactive protein 2, homolog (human) | 2.13 | 0.00052288 |
| 215866 | LOC215866 | hypothetical protein LOC215866 | 2.12 | 0.0013012 |
| 22403 | Wisp2 | WNT1 inducible signaling pathway protein 2 | 2.12 | 0.0064327 |
| 21825 | Thbs1 | thrombospondin 1 | 2.12 | 0.00011145 |
| 12489 | Cd33 | CD33 antigen | 2.12 | 0.0035882 |
| 14972 | H2-K1 | histocompatibility 2, K1, K region | 2.12 | 0.00010236 |
| 27361 | Sepx1 | selenoprotein X 1 | 2.12 | 0.0010017 |
| 210719 | Mkx | mohawk | 2.11 | 0.00093718 |
| 21838 | Thy1 | thymus cell antigen 1, theta | 2.11 | 0.00023721 |
| 54354 | Rassf5 | Ras association (RalGDS/AF-6) domain family 5 | 2.11 | 0.00058134 |
| 14744 | Gpr65 | G-protein coupled receptor 65 | 2.10 | 0.00070573 |
| 17970 | Ncf2 | neutrophil cytosolic factor 2 | 2.10 | 0.00073191 |
| 394434 | Ugt1a9 | UDP glucuronosyltransferase 1 family, polypeptide A9 | 2.10 | 0.00016834 |
| 12267 | C3ar1 | complement component 3a receptor 1 | 2.10 | 6.61E-05 |
| 20302 | Ccl3 | chemokine (C-C motif) ligand 3 | 2.09 | 0.0096658 |
| 226421 | 5430435G22Rik | RIKEN cDNA 5430435G22 gene | 2.09 | 0.00025364 |
| 76044 | Ncapg2 | non-SMC condensin II complex, subunit G2 | 2.09 | 5.35E-05 |
| 27056 | Irf5 | interferon regulatory factor 5 | 2.09 | 0.00014541 |
| 12491 | Cd36 | CD36 antigen | 2.09 | 0.00042079 |
| 20568 | Slpi | secretory leukocyte peptidase inhibitor | 2.09 | 0.0068717 |
| 17381 | Mmp12 | matrix metallopeptidase 12 | 2.09 | 0.001043 |
| 13601 | Ecm1 | extracellular matrix protein 1 | 2.08 | 0.0013764 |
| 66815 | Ccdc109b | coiled-coil domain containing 109B | 2.08 | 0.00057799 |
| 15162 | Hck | hemopoietic cell kinase | 2.08 | 0.00043688 |
| 16186 | Il2rg | interleukin 2 receptor, gamma chain | 2.07 | 0.00020678 |
| 20354 | Sema4d | sema domain (semaphorin) 4D | 2.07 | 0.0001046 |
| 19229 | Ptk2b | PTK2 protein tyrosine kinase 2 beta | 2.07 | 0.00013604 |
| 66889 | Rnf128 | ring finger protein 128 | 2.06 | 0.00012055 |
| 235320 | Zbtb16 | zinc finger and BTB domain containing 16 | 2.06 | 0.0029301 |
| 210293 | Dock10 | dedicator of cytokinesis 10 | 2.06 | 7.83E-05 |
| 18669 | Abcb1b | ATP-binding cassette, sub-family B (MDR/TAP), member 1B | 2.05 | 0.0003205 |
| 109225 | Ms4a7 | membrane-spanning 4-domains, subfamily A, member 7 | 2.03 | 2.95E-05 |
| 16854 | Lgals3 | lectin, galactose binding, soluble 3 | 2.03 | 6.99E-05 |
| 73910 | Arhgap18 | Rho GTPase activating protein 18 | 2.02 | 0.0041448 |
| 74048 | 4632428N05Rik | RIKEN cDNA 4632428N05 gene | 2.02 | 9.38E-05 |
| 16164 | Il13ra1 | interleukin 13 receptor, alpha 1 | 2.02 | 0.00020784 |
| 20698 | Sphk1 | sphingosine kinase 1 | 2.02 | 0.0048216 |
| 110006 | Gusb | glucuronidase, beta | 2.02 | 0.00027011 |
| 16790 | Anpep | alanyl (membrane) aminopeptidase | 2.02 | 0.00013298 |
| 11847 | Arg2 | arginase type II | 2.02 | 0.0039292 |
| 24088 | Tlr2 | toll-like receptor 2 | 2.01 | 0.00054402 |
| 14226 | Fkbp1b | FK506 binding protein 1b | 2.01 | 0.00015333 |
| 66395 | Ahnak | AHNAK nucleoprotein (desmoyokin) | 2.01 | 0.0016287 |
| 228482 | Arhgap11a | Rho GTPase activating protein 11A | 2.01 | 0.00031828 |
| 226844 | 9630055N22Rik | RIKEN cDNA 9630055N22 gene | 2.01 | 9.04E-05 |
| 11717 | Ampd3 | AMP deaminase 3 | 2.00 | 0.00011347 |
| 225655 | Slmo1 | slowmo homolog 1 (Drosophila) | 2.00 | 0.0024715 |
|  |  |  |  |  |
| **Genes downregulated** | |  |  |  |
| 27220 | Cartpt | CART prepropeptide | -5.92 | 0.001504 |
| 14580 | Gfap | glial fibrillary acidic protein | -4.39 | 0.00073191 |
| 69368 | Wdfy1 | WD repeat and FYVE domain containing 1 | -4.15 | 2.91E-06 |
| 66442 | Spbc25 | spindle pole body component 25 homolog (S. cerevisiae) | -3.62 | 0.0084204 |
| 237553 | Trhde | TRH-degrading enzyme | -3.56 | 0.0014715 |
| 12140 | Fabp7 | fatty acid binding protein 7, brain | -3.1 | 1.52E-05 |
| 20512 | Slc1a3 | solute carrier family 1, member 3 | -2.79 | 0.0046863 |
| 14586 | Gfra2 | glial cell line derived neurotrophic factor family receptor alpha 2 | -2.75 | 0.0014513 |
| 14598 | Ggt1 | gamma-glutamyltransferase 1 | -2.66 | 5.36E-05 |
| 18115 | Nnt | nicotinamide nucleotide transhydrogenase | -2.53 | 8.60E-05 |
| 20262 | Stmn3 | stathmin-like 3 | -2.48 | 0.00070396 |
| 19242 | Ptn | pleiotrophin | -2.41 | 0.0066797 |
| 20674 | Sox2 | SRY-box containing gene 2 | -2.4 | 0.0058896 |
| 14763 | Gpr37 | G protein-coupled receptor 37 | -2.36 | 0.0066399 |
| 12971 | Crym | crystallin, mu | -2.35 | 0.0037188 |
| 75858 | Speer7-ps1 | spermatogenesis associated glutamate (E)-rich protein 7 | -2.31 | 0.00033076 |
| 11500 | Adam7 | a disintegrin and metallopeptidase domain 7 | -2.28 | 0.00061525 |
| 67473 | 1300013J15Rik | RIKEN cDNA 1300013J15 gene | -2.28 | 0.001533 |
| 14799 | Gria1 | glutamate receptor, ionotropic, AMPA1 (alpha 1) | -2.26 | 0.0051223 |
| 13618 | Ednrb | endothelin receptor type B | -2.24 | 0.0042803 |
| 18121 | Nog | noggin | -2.17 | 0.0054202 |
| 211739 | Vstm2 | V-set and transmembrane domain containing 2 | -2.12 | 0.0063321 |
| 72599 | Pdia5 | protein disulfide isomerase associated 5 | -2.11 | 0.0015128 |
| 14805 | Grik1 | glutamate receptor, ionotropic, kainate 1 | -2.07 | 0.00021664 |
| 12741 | Cldn5 | claudin 5 | -2.07 | 0.0041074 |
| 76217 | Jakmip2 | janus kinase and microtubule interacting protein 2 | -2.06 | 0.0071753 |
| 319149 | Hist1h3d | histone cluster 1, H3d | -2.06 | 0.0081023 |
| 27028 | Ermap | erythroblast membrane-associated protein | -2.05 | 0.0035709 |
| 74651 | 4930435M08Rik | RIKEN cDNA 4930435M08 gene | -2.04 | 0.004997 |
| 18616 | Peg3 | paternally expressed 3 | -2.04 | 0.00027162 |
| 12576 | Cdkn1b | cyclin-dependent kinase inhibitor 1B | -2.02 | 0.0079249 |
| 20913 | Stxbp4 | syntaxin binding protein 4 | -2.02 | 0.0081845 |
| 11535 | Adm | adrenomedullin | -2.01 | 0.00092885 |
