# Supplementary figures and images for: The Aged Retinal Pigment Epithelium/Choroid: A Potential Substratum for the Pathogenesis of Age-Related Macular Degeneration
Source: PLoS One. 2008 Jun 4;3(6):e2339. doi: 10.1371/journal.pone.0002339 (PMC2394659; doi:10.1371/journal.pone.0002339)

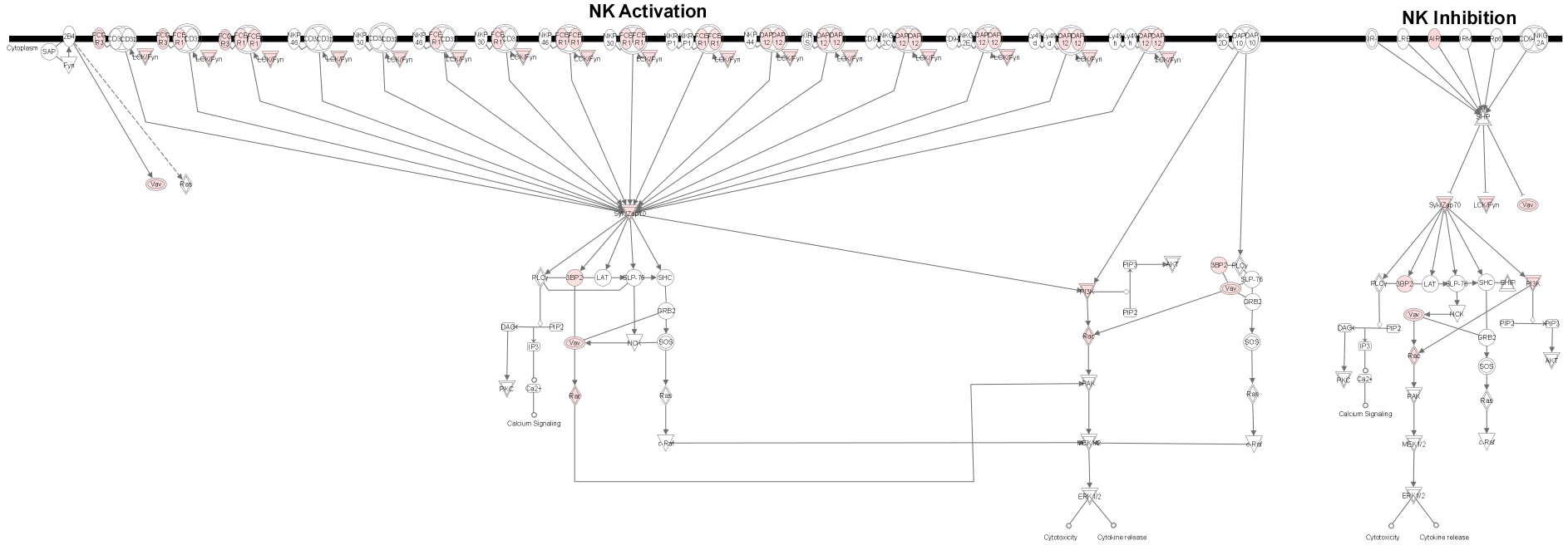

Supplement: Figure S1 — Pathway diagram showing the molecules involved in NK cell signaling and their interaction. The diagram was modified from Ingenuity Pathway Analysis (Ingenuity® Systems). (3.73 MB TIF) [file pone.0002339.s002.tif]

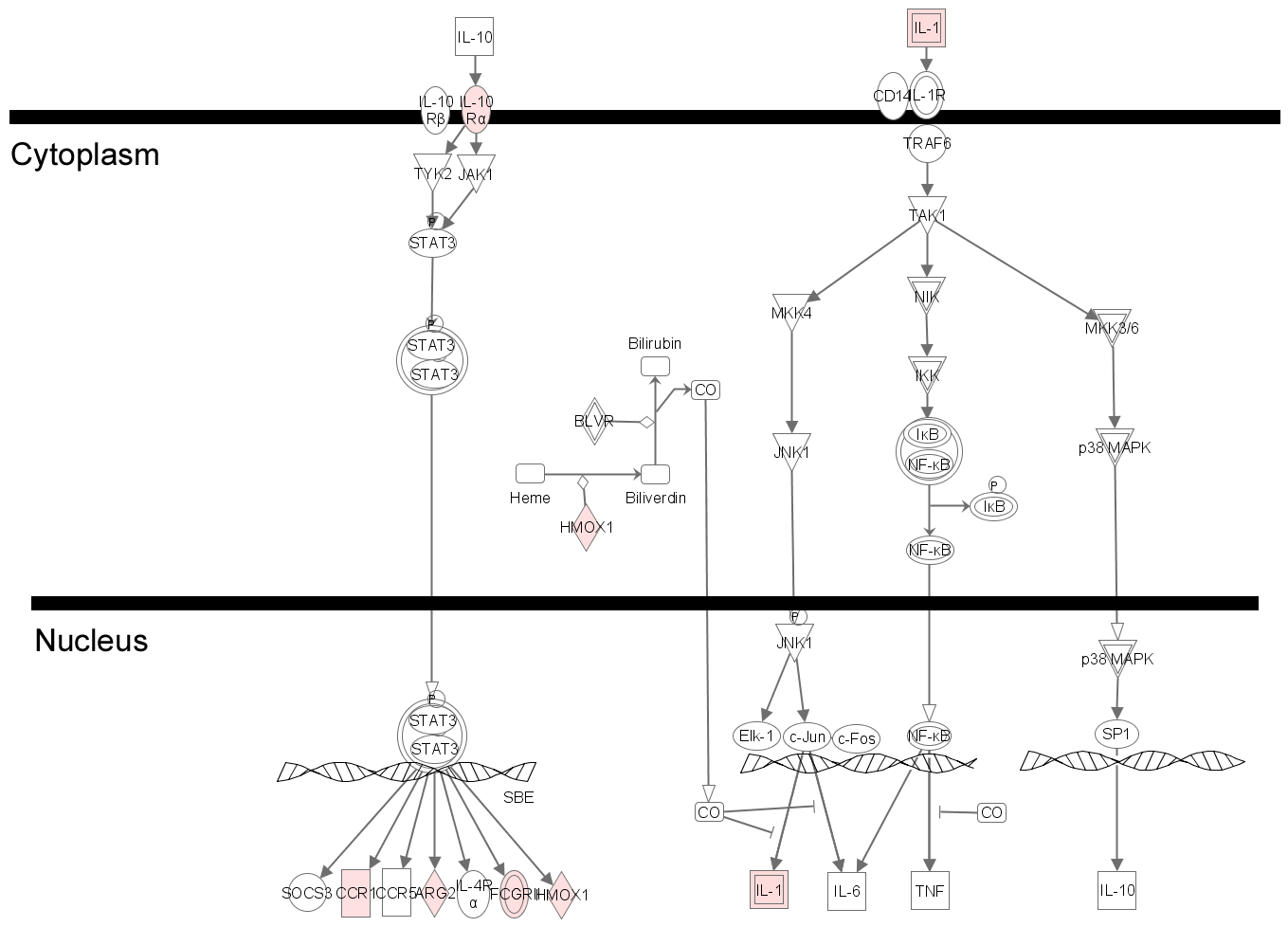

Supplement: Figure S2 — Pathway diagram showing the molecules involved in IL-10 signaling and their interaction. The diagram was modified from Ingenuity Pathway Analysis (Ingenuity® Systems). (9.74 MB TIF) [file pone.0002339.s003.tif]
